# Importation models for travel-related SARS-CoV-2 cases reported in Newfoundland and Labrador during the COVID-19 pandemic

**DOI:** 10.1098/rsos.241902

**Published:** 2025-11-26

**Authors:** Zahra Mohammadi, Monica Gabriela Cojocaru, Julien Arino, Amy Hurford

**Affiliations:** ^1^ University of Guelph, Guelph, Ontario, Canada; ^2^ University of Manitoba, Winnipeg, Manitoba, Canada; ^3^ Department of Biology, Memorial University of Newfoundland, St John's, Newfoundland and Labrador, Canada

**Keywords:** importation modelling, travel measures, SARS-CoV-2, travel-related cases, pandemic, Newfoundland and Labrador

## Abstract

During the COVID-19 pandemic, the World Health Organization updated guidelines for travel measure implementation to recommend consideration of a region's specific epidemiological, health system and socioeconomic context. As such, travel measure implementation decisions require region-specific data, analysis and models to support risk assessment frameworks. From May 2020 to May 2021, the Canadian province of Newfoundland and Labrador (NL) implemented travel measures that required self-isolation and testing of individuals returning from out-of-province travel. We found that during the pandemic travel to NL decreased by 82%. Our best model was 135 times more likely to explain reported travel-related cases arriving in NL than a model where travel volume and infection data did not consider the Canadian jurisdiction of origin. To test an approach used in other studies, we formulated a model without considering the travel-related case data and found that this model performed very poorly. We conclude that importation models need to be supported with data describing the daily number of travel-related cases arriving in Canadian jurisdictions and daily travel volumes originating from each country and each Canadian province and territory. While there was some reporting of this information during the COVID-19 pandemic, these data were not consistently reported or easily accessible.

## 1. Introduction

On 31 January 2020, due to an outbreak of SARS-CoV-2 in Wuhan, China, the World Health Organization (WHO) advised other countries to expect SARS-CoV-2 cases and be prepared for outbreak containment, but measures that would restrict travel and trade were explicitly not recommended [1,2]. State Parties were to notify WHO within 48 h of the public health rationale and justification of measures that would significantly interfere with international traffic [2]. Despite broad consensus prior to the pandemic that during a public health emergency travel measures significantly impacting travel and trade should not be implemented, during the initial phase of the COVID-19 pandemic most countries implemented such travel measures [1,3,4]. The next 2 years saw substantial variation in the implementation and strictness of travel measures between [3] and within [5,6] countries. In July 2021, WHO provided updated recommendations stating that international travel-related measures should be ‘proportionate to the public health risk’ and adapted to a country’s ‘specific epidemiological, health system and socioeconomic context’ and recommended a risk-based approach [7].

The inconsistent implementation of travel measures during the first 2 years of the pandemic may have been due to the low quality of evidence to support policy. Systematic reviews [1,8] report that travel measures may have had a positive impact on infectious disease outcomes, and reduced and delayed imported SARS-CoV-2 cases from Wuhan, but that overall the quality of evidence was low. Most evidence was due to modelling studies with a lack of ‘real-world’ data [8], with inconsistent parameter estimates and assumptions, and that overlooked the impact of undetected cases outside China [1].

To better support modelling studies with ‘real-world data’, and to consider the specific context in which travel measures are applied, our analysis focuses on Newfoundland and Labrador (NL), a Canadian province that implemented travel measures during the COVID-19 pandemic. Our analyses contribute data, parameter estimates and models to support decisions and inform best approaches to reporting during a pandemic. In particular, we note there are few data and guidelines to inform travel measure implementation within countries during a pandemic. We develop models that consider data describing travel volume arriving in NL, infection prevalence at a traveller’s origin and reported travel-related cases in NL. Three studies have previously considered all of these data types [9–11], but many importation modelling studies have had to proceed without all of these data sources available; for example, Godin *et al.* [12] and Steyn *et al.* [13] do not consider travel volumes. We develop different models to determine the impact of data gaps on the reliability of importation models. The data gaps we consider are: incomplete information on travel volumes, no data describing reported travel-related cases in the region of interest, no information on infection underreporting at travellers’ origins and infection prevalence and travel volume data only available for large geographic areas.

Travel volume data are often incomplete. For example, the OpenSky database and the Official Aviation Guide are the travel volume data sources used in Russell *et al.* [14], and these sources report only travellers that arrive by air, and not by land or sea. Exclusion of travellers arriving by some travel modes is just one type of exclusion that occurs in travel volume data sources. Other exclusions can be types of workers or residents of the destination jurisdiction. We corrected the travel volume data for exclusions, and estimated how travel volumes to NL changed during the pandemic and given the enacted travel measures.

Several studies have estimated the epidemiological risk due to imported infections without any data reporting travel-related cases [14–17], and other studies have used data that do not distinguish between travel-related and community cases [18–22]. Data describing travel-related cases in travellers arriving in Canada were not consistently easily accessible to researchers during the pandemic. Post-arrival testing of travellers occurred in Canada, and these travel-related cases were sometimes reported on provincial and territorial public health websites, reported on by the media [23], and compiled into accessible formats by volunteers [24,25]. Travel-related case data could have been used to assess the accuracy of the predictions made in Milwid *et al*. [17] describing the number of infections in international arrivals to Toronto Pearson, Montréal-Trudeau, Vancouver International and Calgary International airports. Travel-related case data could also have been used to assess the accuracy of the number of importations predicted to occur in Atlantic Canada and the territories made by Hincapie *et al.* [20]. These travel-related case data exist [26], but were not considered by Hincapie *et al.* [20], so in §A of the electronic supplementary material we make this comparison.

To replicate the approach to importation modelling when travel-related case data are unavailable [17,20], we formulate a mechanistic model that considers the processes that give rise to travel-related cases, but is not fitted to the travel-related case data. This mechanistic modelling approach estimates parameters from published studies as a method to overcome the limitations arising due to travel-related case data being unavailable. The predictions of this mechanistic model are validated with the travel-related case data, which are completely independent of the mechanistic model’s parametrization. We choose this modelling approach to test whether the predictions of mechanistic models parametrized independently of travel-related case data (i.e. as occurs in [17] and [20]) are accurate and to provide evidence for why reporting of travel-related cases is necessary during a pandemic. We also test whether importation models are less reliable if travel volume and infection prevalence data are aggregated as Canada or international, rather than considering each country, and Canadian province or territory of origin to inform the types of data that are needed to model importations and support decisions to implement travel measures during a public health emergency.

## 2. Methods

### 2.1. Background

NL (population: 510 550 [27]) is the second smallest Canadian province and has few points of entry. Most non-resident travellers to NL visit the island of Newfoundland (93% [28]; population: 483 895 [27]) and arrive by air to St John’s International Airport. From 4 May 2020 to 30 June 2021, the government of NL implemented travel measures that required non-residents to complete Travel Declaration Forms (TDFs) and self-isolate for 14 days after arrival. Rotational workers, NL residents working in other provinces, are a significant proportion of the NL workforce [29] and during the COVID-19 pandemic were subject to specific self-isolation requirements and testing regimes.

### 2.2. Data overview

#### 2.2.1. Travel volume to Newfoundland and Labrador

We consider three data sources that report travel volumes: International Air Transit Authority (IATA) flight passenger data; TDFs completed by non-NL residents and other non-exempt individuals upon arrival to NL during the COVID-19 pandemic; and Frontier Counts (FC; Statistics Canada 2020–2021 [30]) completed at the Canadian border. The three sources of travel volume data have some overlap and different limitations (table 1), and when combined with tourism surveys from the Government of Newfoundland and Labrador (2020–2021) [31], we were able to estimate correction factors to determine the ‘total travel volume’ arriving in NL from January 2019 to March 2020 and September 2020 to May 2021 (electronic supplementary material, §B.6), where ‘total travel volume’ includes arrivals by air, sea and land ports of entry, and all traveller types including crew members, NL residents and rotational workers. After estimating the total travel volume, we then stratified arriving travellers as regular travellers or rotational workers, and by travel origins. For travellers arriving from Canada, the origin can be any of the nine provinces (excluding NL) and the territories, where the three territories are considered as one because there is insufficient information on infection underreporting to separate each territory. For travellers arriving from international origins, we considered the eight countries comprising the most arrivals to NL and aggregated all remaining travellers into an ‘other countries’ category (see §B.5 of the electronic supplementary material for details). The provincial and federal travel measures that applied to travellers arriving in NL during the pandemic are summarized in table 2.

**Table 1.** Limitations of travel volume data sources. IATA (s=1), TDF (s=2) and FC (s=3) report an origin (either Canada or international), but report travel volumes that exclude some travellers that might spread infections to NL residents. When exclusions or exemptions apply to particular travel modes (air, sea or land) or traveller types (i.e. crew or NL residents), the value of the exclusions indicator variable, 1MODES( or 1TYPE(, is 1; and the indicator variable is 0 if this exemption does not apply. These indicator variables appear in equation (B1), and the magnitude of the correction for the exclusion is given in table B1 (§B in the electronic supplementary material). The travel origin in TCAR reports (s=4) was not reported, but the information in these reports was used to estimate the magnitude of the exclusions for the other data sources.

*s*	data source	time period	pandemic	travel modes excluded	$1_{\text{MODES}}=$	origin	traveller type exclusions	$1_{\text{TYPE}}=$
1	International Air Transport Authority (IATA)	January 2019–March 2020 (monthly)	before	land, sea	1	Canada and international	Canadian crew	1 if Canada, 0 if international
2	Travel Declaration Forms (TDF)	September 2020–May 2021 (daily)	during	none	0	Canada and international	NL residents, crew, and other exempt travellers	1
3	Frontier Counts (FC)	January 2019–May 2021 (monthly)	before and during	none	0	international	none	0
4	Department of Tourism, Culture, Arts, and Recreation (TCAR)	January 2019–December 2021 (monthly)	before and during	none	N/A	unknown	NL residents	N/A

**Table 2.** Federal (Canada) and provincial (NL) travel measures from September 2020 to May 2021 [32]. ‘Line’ corresponds to the numbering in figure 1A.

line	dates	measure	level
1	13-03-2020	cruise ship season postponed	federal
	14-03-2020	14 days self-isolation required for individuals returning from international travel	provincial
	20-03-2020	14 days self-isolation required for individuals returning from out-of-province travel	provincial
2	04-05-2020	travel declaration forms and self-isolation plan required for non-NL resident entry to NL	provincial
3	09-06-2020	relaxation of travel measures for foreign nationals with immediate family in Canada	federal
4	03-07-2020	Atlantic bubble: no self-isolation requirement for residents of Prince Edward Island, New Brunswick and Nova Scotia	provincial
5	31-08-2020	relaxation of travel measures for non-Atlantic Canada residents who own a second home or cabin in NL	provincial
6	20-10-2020	relaxation of travel measures for international students attending institutions with a COVID-19 readiness plan	federal
7	26-11-2020	Atlantic bubble suspended	provincial
8	01-02-2021	all international passenger flights must land at either the Vancouver, Calgary, Toronto or Montréal airports	federal
9	27-03-2021	passengers on provincial ferries limited to 50% of capacity	provincial

#### 2.2.2. Infection prevalence at travellers’ origin

We estimate infection prevalence at the travellers’ origin from daily incidence in the Canadian provinces and territories as reported by the Public Health Agency of Canada (Canada) and John Hopkins University (international) [33] and adjusted for reporting delays as this information is used for explanatory variables in statistical models. We multiplied daily reported infections by an underreporting coefficient based on seroprevalence data reported by the COVID Immunity TaskForce (Canada) and using the approach described in [34] (international; see §C in the electronic supplementary material for complete details). We estimate infection prevalence as a proportion by dividing by the population size.

**Figure 1.**
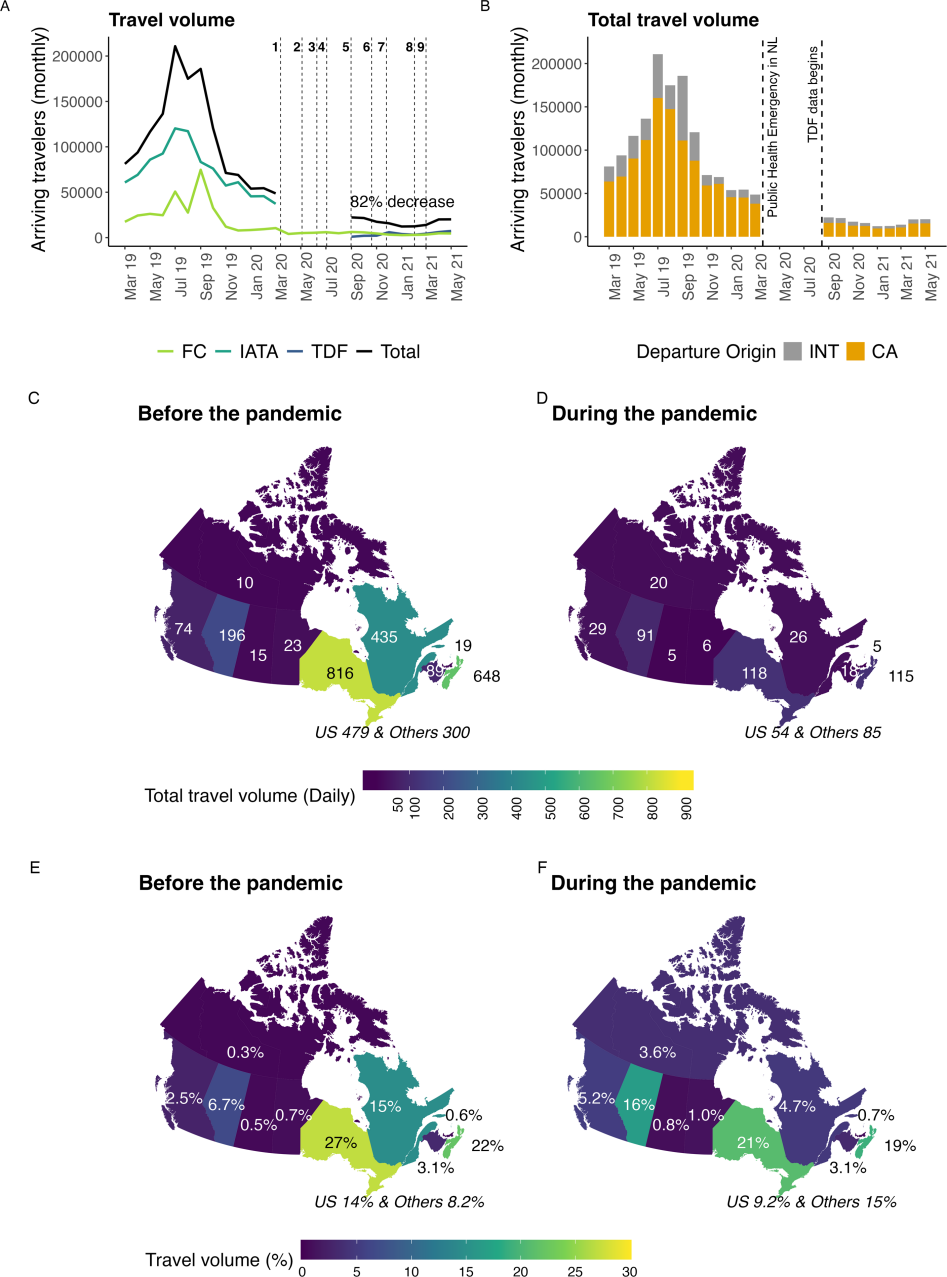
(A) The total travel volume arriving in NL before and during the pandemic (dark purple line, equation (B1) in the electronic supplementary material), and travel volumes reported by individual data sources: IATA (dark green line); TDF (blue line); and FC (light green line). The numbered vertical lines correspond to travel measures implemented in NL (table 2). (B) Total travel volume arriving from Canadian and international origins. (C,D) The average number of travellers arriving daily before and during the pandemic and their region of origin, where these averages are taken over nine months. (E) The average percentage of arrivals from particular origins for nine months during the pandemic (September 2019 to March 2020 and April to May 2019) and (F) for the same nine months during the pandemic (September 2020 to May 2021).

#### 2.2.3. Travel-related cases reported in Newfoundland and Labrador

Daily travel-related cases of Canadian and international origin were obtained from Newfoundland and Labrador Health Services—Digital Health (figure B2 in the electronic supplementary material; Newfoundland and Labrador Health Research Ethics Board reference number 2021.013), but were also reported publicly in Public Service Announcements from the NL Department of Health and Community Services during the pandemic. In NL, close contacts of travel-related cases were required to undergo asymptomatic testing, and if positive were reported as ‘close contacts of travellers’, and were not included in the reported number of travel-related cases. In this article, travel-related cases arise from imported cases that test positive.

### 2.3. Modelling overview

#### 2.3.1. Statistical importation models

We formulated statistical models with a linear model structure. The statistical models were generalized linear models with a negative binomial or Poisson error distribution and where the response variable is the daily travel-related cases reported in NL either from Canada or from international origins. The statistical models have explanatory variables that are daily total travel volume from an origin (§2.2.1), daily infection prevalence at origin (§2.2.2) or both these variables and interaction terms. For models to predict daily travel-related cases of Canadian origin, we considered stratification of all model variables for each Canadian province and the territories, and aggregation of variables for all of Canada. For models to predict daily travel-related cases of international origin, we consider stratification of all model variables for eight common countries of origin with all other countries aggregated as ‘other international’, and an aggregated model with all non-Canadian countries of origin combined. Models were fitted using glm.nb from the MASS R package [35] and prediction intervals were generated using add_pi from the ciTools package [36].

#### 2.3.2. Model without travel-related case data

Using a modelling approach similar to that of Milwid *et al*. [17] and Hincapie *et al.* [20], we consider the epidemiology of infection importation to predict the number of travel-related cases expected to be reported each day in NL from September 2020 to June 2021. The processes we consider include travel volumes, infection prevalence at travellers’ origin, days since exposure for infected travellers and testing policies in NL (table D5 in the electronic supplementary material contains descriptions of variables and parameters). Unlike the statistical models which are fitted to the travel-related case data, this independently parametrized mechanistic model is developed based on the processes typically represented in mechanistic importation models, with the number of travel-related cases predicted based on the testing of travellers that occurred in NL during the pandemic. Figure D3 in the electronic supplementary material is an overview of the mechanistic model.

For travellers arriving in NL, polymerase chain reaction tests were mandatory for anyone symptomatic, for travellers that were passengers on flights for which exposure notifications were issued, and for rotational workers upon their return to NL (described in tables D6 and D7 of the electronic supplementary material). For travellers from Canadian origins, the predicted number of travel-related cases reported in NL at t is


(2.1) $$R_{CA}(t)=\sum_{i}R_{i}^{r,e}(t)+R_{i}^{r,s}(t)+R_{i}^{rw}(t),$$

where the sum i is across all Canadian provinces (except NL) and the territories, $R_{i}^{r,e}(t)$ is the number of regular travellers that are predicted to test positive and are tested due to an exposure notification, $R_{i}^{r,s}(t)$ is the number of regular travellers that are predicted to test positive and requested a test because they developed symptoms post-arrival and $R_{i}^{rw}(t)$ is the number of rotational workers that are predicted to test positive on at least one mandatory post-arrival test.

For travellers from international origins, the predicted number of reported travel-related cases in NL is


(2.2) $$R_{INT}(t)=\sum_{i}R_{i}^{r,e}(t)+R_{i}^{r,s}(t),$$

which is similarly defined as equation (2.1) except that the sum is across different countries of origin, i, and only regular travellers are considered in the sum because by definition rotational workers do not return from international origins.

#### 2.3.3. Assessment of model fit

Model fit was assessed by calculating the negative log likelihood (nLL). The likelihood ratio test statistic is calculated as


(2.3) $$LR=-2\left({nLL}_{0}-nLL\right),$$

where nLL${,}_{0}$ is a null model (or ‘constant only’ model) with the only fitted parameters the intercept, $\beta_{0}$, and the overdispersion parameter, θ, when the negative binomial error distribution is considered (see equations (E22) and (E25)). The likelihood ratio test statistic was not calculated for the models that were not fit to travel-related case data (i.e. the mechanistic model) because these models were not nested.

The accuracy of the predictions for the model that did not consider the travel-related case data is assessed by calculating the likelihood of $R_{\mathrm{CA}}(t)$ given the reported number of travel-related cases of Canadian origin ($Y_{t,\mathrm{CA}}$), and the likelihood of $R_{\text{INT }}(t)$ given the reported number of travel-related cases of international origin ($Y_{t,\text{ INT }}$), and assuming a Poisson error distribution, i.e.


(2.4) $$Y_{t,j}∼\mathrm{POISSON}\left(R_{j}(t)\right).$$

The Poisson distribution was used because it is not possible to fit an overdispersion parameter as the mechanistic model predicts only the mean number of reported travel-related cases and not the variance.

The best model is defined as the model with the smallest nLL. As the aim of our study is to most accurately model importations, the reliability of the model is the only relevant consideration; however, our best models did have the most parameters and for context we also report the corrected Akaike information criteria (AICc) scores. Goodness of fit for predictions made by the best model was assessed by calculating the residual deviance and testing the null hypothesis that the predictions of the fully saturated model and the best model were different.

Data and relevant code for this research work are stored in a GitHub repository (https://github.com/ahurford/importation-revision) and have been archived within the Zenodo repository [37].

## 3. Results

We found that the total travel volume arriving in NL declined by 82% during the pandemic while travel measures were enacted (September 2020 to May 2021) compared to the same period a year prior (figure 1A; dark purple line). Relative to the same nine months 1 year prior to the pandemic, the average percentage of travellers arriving in NL increased from British Columbia (2.5 to 5.2%), Alberta (6.7 to 16%), the Canadian territories (0.3 to 3.6%) and international origins not the United States (8.2 to 15%; figure 1E,F). The average percentage of travellers arriving in NL during the pandemic decreased from Ontario (27 to 21%), Québec (15 to 4.7%) and the United States (14 to 9.2%; figure 1E,F).

**Figure 2.**
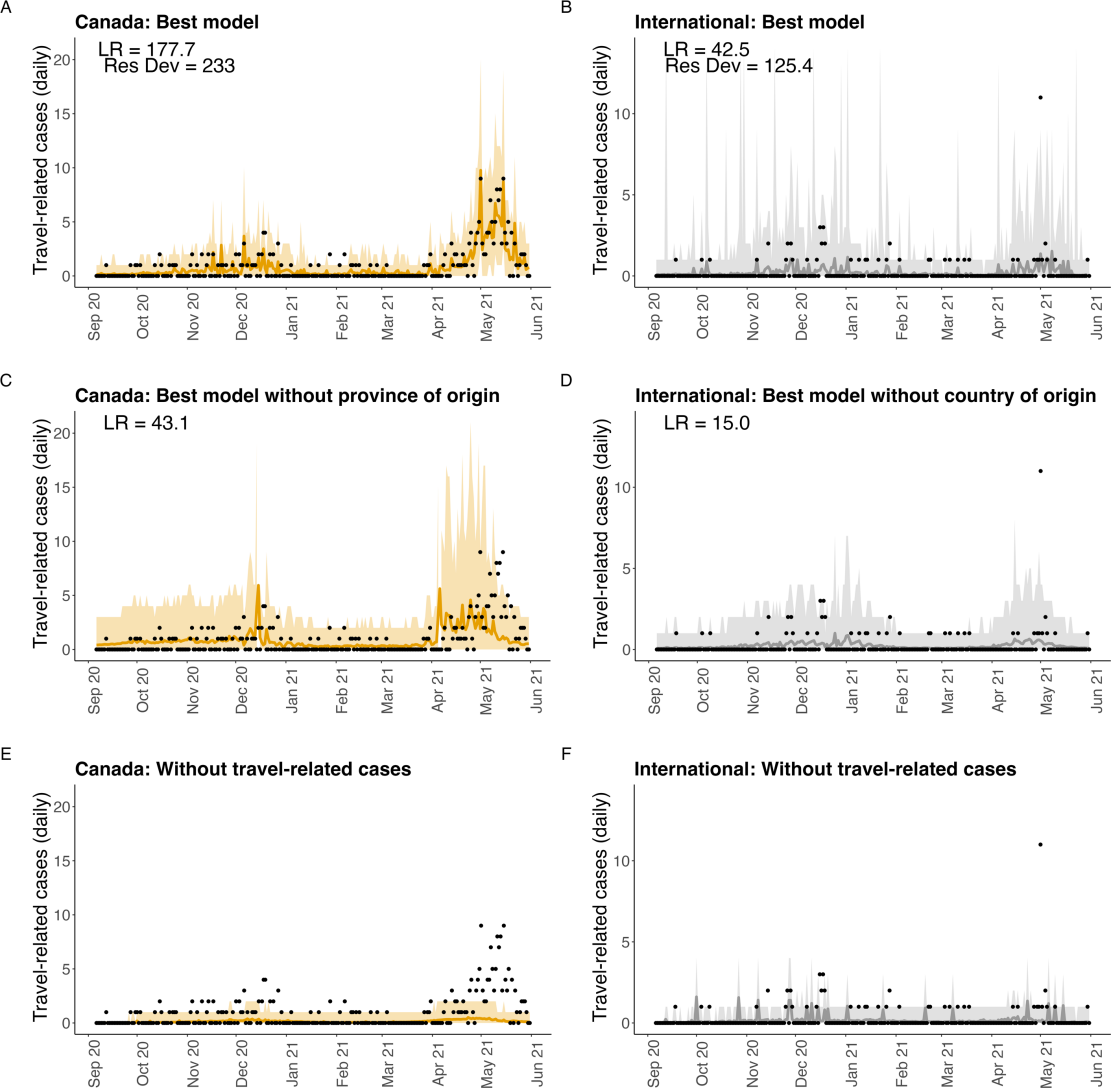
Reported travel-related cases as predicted by the best model (A,B), the best model with explanatory variables aggregated as Canada or international (C,D), and the model that was formulated independently of the travel-related case data (E,F) (see table 3 for the models). The shaded region is a 95% prediction interval and the travel-related case data are shown as black dots.

Our best statistical models had all explanatory variables and interaction terms, where all variables are stratified for each of the 10 possible Canadian jurisdictions of origin, and nine international regions of origin. The best model predicting travel-related cases arriving in NL from Canada (figure 2A) was 177.7 times more likely to explain the data than a null model consisting of only an intercept (table 3). The residual deviance for the best model was 233 with 237 d.f. The chi-squared test found that the null hypothesis that the data are different from the model predictions could not be rejected (p=0.56). All the best models reported in table 3 assume a negative binomial error distribution except the ‘travel volume × infection prevalence (provinces)’ model, which could not be fitted with a negative binomial error distribution and instead assumes the Poisson error distribution (see electronic supplementary material, §E.1, for further details).

**Table 3.** Model fit for predicted daily travel-related cases reported in NL from Canadian and international origins. Models where variables are stratified for Canadian provinces and the territories are denoted with ‘provinces’ in parentheses. Models were variables are aggregated for the Canadian provinces and the territories or for all international origins are denoted with ‘aggregated’ in parentheses. The number of parameters that are fitted is K.

Canada	nLL	LR	*K*	AICc
travel volume × infection prevalence (provinces)	244.2	177.7	31	559.1
infection prevalence (provinces)	270.8	124.8	12	566.8
travel volume (provinces)	294.8	76.8	12	614.9
travel volume × infection prevalence (aggregated)	311.7	43.1	5	633.6
travel volume (aggregated)	321.4	23.6	3	648.9
infection prevalence (aggregated)	326.6	13.1	3	659.4
constant only	333.2	0	2	670.5
without travel-related cases	538.2	not nested	0	1076.5

international				
travel volume × infection prevalence (countries)	131.4	42.5	28	325.7
infection prevalence (countries)	143.0	19.4	10	306.8
travel volume × infection prevalence (aggregated)	145.2	15.0	5	300.6
infection prevalence (aggregated)	145.3	14.7	3	296.7
travel volume (countries)	147.0	11.2	11	317.1
travel volume (aggregated)	151.8	1.8	3	309.7
constant only	152.7	0	2	309.4
without travel-related cases	191.2	not nested	0	382.4

The best model that aggregated the explanatory variables without distinguishing the Canadian jurisdiction of origin was 43.1 times more likely to explain the data than the ‘constant only’ model consisting of only an intercept (figure 2C). As such, the model that was stratified to consider each of 10 Canadian jurisdictions of origin was 134.6 times more likely to explain the data than the best aggregated model. The mechanistic model that was not fitted to the travel-related case data yielded worse predictions (nLL = 538.2; figure 2E) than the model consisting of only an intercept and assuming a Poisson error distribution (nLL = 407.0; table E8).

Travel-related cases arriving in NL from international origins were best predicted by a statistical model with all explanatory variables and interaction terms, and where all variables were stratified by each of nine possible origins (figure 2B). The best model was 42.5 times more likely to explain the data than the ‘constant only’ null model (table 3). The residual deviance of the best model was 125.4 with 241 d.f. The chi-squared test found that the null hypothesis that the data are different from the model predictions could not be rejected (p=1). When country-level data were not available, the best model was 15.0 times more likely to explain the data than a constant model. The model that was not fitted to the travel-related case data yielded worse predictions (nLL = 191.2; figure 2F) than the model consisting of only an intercept and assuming a Poisson error distribution (nLL = 178.7; table E8). The fitted coefficients for the best models are reported in §E.1 of the electronic supplementary material.

Only the TDF travel volume data source reports daily travel volume, so to determine the effect of using just one travel volume data source it was necessary to fit to the travel-related case data aggregated by month (nine observations). Results are reported in table E9 of the electronic supplementary material; however, generally, the lack of daily travel volume estimates (only one per month) reduced our ability to formulate and test the models. We also found that correcting infection prevalence for underreporting had only a small effect on model fit (table E10 in the electronic supplementary material).

## 4. Discussion

In July 2021, the WHO updated guidelines for international travel [7] to advise that local epidemiology, and public health and health system performance and capacity should be considered to determine if travel measures are appropriate during a pandemic. Analysis of regional data and regionally specific models are needed because decisions of whether travel measures are appropriate depend on regional characteristics.

We estimated that travel measures (described in §B.2 of the electronic supplementary material) reduced the total travel volume arriving in NL by 82% (September 2020–May 2021) compared to the same nine months prior to the pandemic (figure 1A, dark purple line). This finding is similar to another study finding an estimated 79% reduction in non-resident visitor volume to NL for 2020 compared to 2019 [38]. In other countries, air travel is estimated to have declined, on average, 63% for May 2020 (during the pandemic) as compared to May 2019 (before the pandemic [14]). The more substantial reduction in travel to NL may have been due to stricter travel measures for entry to NL than for other countries and regions during the study period. Most notably, non-NL residents entering NL from other regions of Canada were required to complete TDFs and have a self-isolation plan to submit to a government representative at entry. These results help quantify the impact of travel measures implemented during a pandemic, which is necessary since modelling that predicts clinical cases depends on assumptions describing how travel measures affect travel volume [39].

We found that during the pandemic, there was a 10% increase in arrivals from Alberta and a 10% decrease in arrivals from Québec to NL (figure 1E,F). Evidence for this result can be seen in table B3 of the electronic supplementary material, where the percentages before the pandemic (January 2019–March 2020) are calculated from the IATA data, and the percentages during the pandemic (September 2020–May 2021) are calculated from the TDF data. This shift in the percentage of travellers arriving from different provinces may be explained by the high percentage of NL’s rotational workers that work in Alberta (57%) as compared to Québec (2%; table B3 in the electronic supplementary material), while the travel volume of regular travellers from Québec before the pandemic is relatively high (11–26%, third highest behind Ontario and Nova Scotia; table B3 in the electronic supplementary material). Rotational workers likely continued working during the pandemic, while regular travellers may have delayed or cancelled trips. Importation models used during public health emergencies often consider travel volume data. An implication of our findings is that the travel volume data used for these calculations need to be reported daily, otherwise the type of importation modelling that can be done is very limited (i.e. cannot be stratified by province and territory of origin), and that travel volume data collected before the pandemic may not accurately represent the proportion of travellers arriving from different travel origins. It is necessary to consider travel volume and infection prevalence estimates that are stratified by Canadian province and territory of origin as when these data are aggregated as ‘Canada’, the ability of the model to explain the data is substantially reduced.

Our results find that mechanistic importation models formulated independently of travel-related case data [17,20] are much less reliable than the modelling that can be done, as either mechanistic or statistical models, when travel-related case data are available. The mechanistic modelling approach is to mathematically describe the processes that give rise to imported cases, and to estimate parameters from the published literature. The predictions of some mechanistic models [14,15,20,40] have not been tested or validated, and so the accuracy of this modelling approach is not known. It is perhaps unsurprising that the mechanistic models formulated without travel-related case data performed very poorly, yet travel-related case data are often unavailable to researchers who could use these data in their analyses. The aim of our study was to demonstrate that travel-related cases should be reported during a public health emergency because importation modelling is unreliable without these data. In that the constant-only model performs better than these mechanistic models, travel-related case data are the most important data needed to support importation modelling because even a model with no explanatory variables is better than modelling that occurs without access to travel-related case data (table 3). Without travel-related case data, the only type of modelling that is possible is mechanistic modelling, but mechanistic models can be fitted to data, and if this had been done, most likely the mechanistic model would have performed similarly to the statistical models. As such, the mechanistic model performs poorly not because it is mechanistic, but because it is not fit to the travel-related case data.

Figure F4 in the electronic supplementary material shows that it is plausible that the mechanistic model could fit the data better. The mechanistic model substantially underestimates travel-related cases (figure 2E,F), and this could be because our data sources underestimate travel volumes and/or infection prevalence at travellers’ origin. This suggests that the mechanistic modelling could be improved with better travel volume data or recommending investment in better surveillance to determine infection prevalence. We found that information on underreporting did not improve our statistical models (§E.2.2 in the electronic supplementary material), but accurate information on underreporting could be critical for mechanistic models (§F in the electronic supplementary material). This conclusion further underscores the importance of travel-related case data, as we would not know that the mechanistic model was a substantial underestimate without being able to compare its predictions to the travel-related case data.

The processes that are described by mechanistic importation models may sound compelling, but there are a number of reasons why the assumptions of such models may not hold. Many studies consider only air travel volumes [14,18,20,22,40], although travellers are likely to arrive via other travel modes and this proportion may change seasonally. No one travel volume data source reports the epidemiologically relevant travel volume due to their scope and exemptions. Travel documents completed at international borders omit travellers that originate from within the country, and not all travel between countries requires such documents to be completed (i.e. travel between countries in the European Union). Provinces may conduct surveys to understand tourist preferences, but such surveys (i.e. Government of Newfoundland and Labrador 2020–2021 [31]) usually focus only on non-resident travel, while returning residents and workers are a potential source of imported infection. We combined four data sources (§B in the electronic supplementary material) to overcome limitations of each travel volume data source and to estimate total travel volume; however, this was time consuming, and to model importations urgently during a public health emergency, it is necessary that daily total travel volume data be available in real time.

While our best statistical models fit the reported travel-related case data well (figure 2), the focus of our study was to understand the impacts of data gaps rather than to find the best importation model. The best importation modelling approach has been studied by others. Arnold *et al*. [9] perform statistical modelling to describe the number of cases reported in international travellers isolating in government-managed quarantine facilities in New Zealand during the COVID-19 pandemic. In Arnold *et al*. [9], countries of origin that have low numbers of arrivals, low numbers of cases, or both, are referred to as ‘low information countries’. In our study, the differences between the best statistical model’s predictions and the reported travel-related cases in NL are likely due to low numbers of cases and low travel volumes, particularly for travel-related cases of international origin. Nonetheless, the model fits shown in figure 2 demonstrate that our model predictions have good correspondence with reported travel-related cases.

Regarding forecasting of imported infections arriving in a jurisdiction during a pandemic, the appropriate modelling approach depends on data availability. If travel-related case data reported in the destination jurisdiction are available, then statistical modelling is a good option. However, even when travel-related case data are available, it is good practice to develop both a more mechanistic model and statistical models, to contribute to our knowledge of how to best model importations, to better understand the processes that predict imported infections and so we can avail of the strengths of each approach. A noted strength of mechanistic models, which is less reasonable for statistical models, is the ability to consider counterfactual scenarios [41]. Furthermore, while our analysis has demonstrated that travel-related case data, and travel volume and infection prevalence data available at the country, province and territory level are necessary for reliable importation modelling, we did not have data that linked travel-related cases to travellers’ origin. The availability of these linked data would further support the development and assessment of importation models to support public health decision making during a pandemic and some of this information was available in Canada during the pandemic as was reported in the media [23]. However, when information is reported to the media it is not as easily accessible to researchers as data describing reported cases, vaccination levels, and variant prevalence, which were available for download from the Public Health Agency of Canada’s website.

Testing of travellers at points of entry is necessary to support early warnings for changes in epidemiological patterns [42]. Early in the COVID-19 pandemic, WHO situation reports described transmission classifications as imported cases only, sporadic cases, clusters of cases, local transmission and community transmission, where this information was self-reported by the State Parties [43,44]. We do not consider it necessary for all regions to report the number of cases with a travel history (see also [45]), but reporting of data describing the number of travel-related cases in a jurisdiction that is not experiencing widespread community transmission is necessary to support the development and refinement of importation models, and the results of our study definitively demonstrate this point. Generally, data, models and analysis are needed to inform decisions to implement travel measures in a region, and our results aim to support decision making and inform future approaches to model development.

## Data Availability

Data and relevant code for this research work are stored in GitHub: https://github.com/ahurford/importation-revision, and have been archived within the Zenodo repository [37]. Newfoundland and Labrador Health Services—Digital Health, formerly the Newfoundland and Labrador Centre for Health Information (NLCHI), is the data custodian for the Travel Declaration Form data and Newfoundland and Labrador COVID-19 cases. Some data are reported in the electronic supplementary material [46].
